# Capmatinib for the treatment of *MET*ex14 skipping non-small cell lung cancer: retrospective analysis of real-world data from patients receiving compassionate use treatment in Italy

**DOI:** 10.1007/s10238-025-01934-2

**Published:** 2025-11-18

**Authors:** Fabiana Letizia Cecere, Ettore D’Argento, Francesco Gelsomino, Francesco Pesola, Paolo Bironzo, Salvatore Grisanti, Laura Bonanno, Gianluca Spitaleri, Diletta Valsecchi, Ilaria Marcon, Diego Cortinovis

**Affiliations:** 1 Medical Oncology 2, IRCCS Regina Elena National Cancer Institute Rome, Rome, Italy; 2 Comprehensive Cancer Center, Medical Oncology Department, Fondazione Policlinico, Universitario Agostino Gemelli IRCCS, Rome, Italy; 3 Medical Oncology, IRCCS Azienda Ospedaliero-Universitaria di Bologna, Bologna, Italy; 4 Thoracic Oncology Unit, IRCCS Istituto Tumori “Giovanni Paolo II”, Bari, Italy; 5 Department of Oncology, University of Torino, AOU S. Luigi Gonzaga, Orbassano, Italy; 6 Medical Oncology, ASST Spedali Civili di Brescia, University of Brescia, Brescia, Italy; 7 Department of Surgery, Oncology and Gastroenterology, University of Padova, Padua, Italy; 8 Medical Oncology 2, Istituto Oncologico Veneto IOV I.R.C.C.S, Padua, Italy; 9 Thoracic Oncology Division, European Institute of Oncology, Via Ripamonti, 435, 20141 Milan, Italy; 10 Novartis Farma SpA, Milan, Italy; 11 Medical Oncology Fondazione, IRCCS San Gerardo dei Tintori Monza, Via Pergolesi 33, 20900 Monza, Italy; 12 Medicine Department University Milano-Bicocca, Milan, Italy

**Keywords:** Capmatinib, MET, *MET*ex14 skipping, Non-small cell lung cancer, Oncogenic driver, Targeted therapy

## Abstract

In patients with advanced non-small cell lung cancer (NSCLC), *MET* exon 14 (*MET*ex14) skipping mutations are rare and are associated with a poor prognosis. Capmatinib, a selective oral MET inhibitor, was recently approved for the treatment of advanced *MET*ex14 skipping NSCLC. However, data from real-world experiences are limited. This was a retrospective analysis using real-world data from Italian patients with advanced *MET*ex14 skipping NSCLC who had received at least one treatment with immunotherapy and/or platinum-based chemotherapy and received compassionate use treatment with capmatinib. Patient characteristics, treatment details, discontinuations, and adverse events are reported. Overall, 53 patients received capmatinib after receiving treatment with immunotherapy and/or platinum-based chemotherapy in Italy. Patients had a median age of 74 years, mostly metastatic disease (92.5% of patients), and previously received one (66.0%) or more (2: 28.3%; 3: 5.7%) lines of standard treatment (immunotherapy and/or platinum-based chemotherapy). Most patients (98.1%) started treatment with capmatinib at the full recommended dose of 400 mg twice daily and 77.4% did not require a dose reduction. Twenty-four patients (45.3%) discontinued treatment; the most frequent reason for discontinuing treatment was disease progression, with a median estimated time to treatment discontinuation of 15.2 months. This real-world retrospective analysis confirms that capmatinib is a valuable treatment option for difficult-to-treat *MET*ex14 skipping NSCLC, in agreement with the data from the registration trial.

## Introduction

Lung cancer is a disease with high mortality and is the leading cause of cancer-related death worldwide [1, 2]. In Italy, the number of deaths due to lung cancer was estimated to be 35,700 (23,600 men and 12,100 women) in 2022 [3]. Non-small cell lung cancer (NSCLC) is the most common type of lung cancer [4]. The identification of driver oncogenic variants and the development of therapies that specifically target these alterations have transformed the diagnosis and treatment of advanced NSCLC [1]. Several actionable oncogenic variants are known, and tumor molecular profiling has become an essential component of treatment decisions in NSCLC [1, 5].

The *MET* gene encodes for a transmembrane receptor tyrosine kinase activated by hepatocyte growth factor (HGF), which is involved in cell growth, development, organogenesis, and wound healing [6, 7]. MET oncogenic alterations identified in NSCLC include *MET* exon 14 (*MET*ex14) skipping mutations, gene copy number gain or amplification, and MET protein overexpression [8]. *MET*ex14 skipping mutations result in the deletion from the transcript of a domain implicated in the internalization and degradation of the receptor complex, leading to sustained activity of the MET-HGF signaling pathway, aberrant cell proliferation, and NSCLC tumor growth [9, 10]. *MET*ex14 skipping mutations are relatively rare and estimated to occur in 3–4% of patients with NSCLC [11–13]; they are associated with a poor prognosis, a modest response to standard treatments including immunotherapy, and short progression-free survival (PFS) [14–16]. A retrospective analysis in 147 patients with *MET*ex14-altered lung cancers of any stage (median age 73 years; 74% with adenocarcinoma histology; 69% with metastatic disease) found that 63% were programmed death-ligand 1 [PD-L1] positive and that the tumor mutational burden was lower compared with unselected NSCLC patients [14]. Overall, the response rate to immunotherapy was low (17%) and duration of response and PFS were short.

Two therapies targeting *MET*ex14 skipping mutations—capmatinib and tepotinib—have become available in recent years [17–20]. Capmatinib is a potent, selective, small-molecule MET inhibitor, which is administered orally and can cross the blood–brain barrier [10, 21]. Capmatinib was approved in May 2020 by the United States (US) Food and Drug Administration (FDA) for use in any line of treatment for NSCLC; notably, the label requires that the mutation leading to *MET*ex14 skipping is detected by an FDA-approved test [19]. By contrast, the European Medicines Agency (EMA) granted marketing authorization to capmatinib in June 2022 for the treatment of patients with NSCLC harboring *MET*ex14 skipping mutations “who require systemic therapy following prior treatment with immunotherapy and/or platinum-based chemotherapy” [17]. In May 2023, the Italian Medicines Agency (AIFA) granted the same indication to capmatinib as the EMA [22]. The efficacy and safety of capmatinib were evaluated in GEOMETRY mono-1, a phase 2 study in 364 patients with advanced NSCLC with a *MET*ex14 mutation or *MET* amplification [13, 23]. Among patients with *MET*ex14 skipping mutations, overall response rates (ORR) were 44% and 68% in patients previously treated with one or two lines of therapy and in treatment-naïve patients, respectively; the associated median durations of response were 9.7 months (95% confidence interval [CI] 5.6–13.0) among previously treated patients and 12.6 months (95% CI 5.6 to not estimated) among treatment-naïve patients [23]. Overall, 98% of patients experienced adverse events (AEs); the most frequently reported AEs with capmatinib were low-grade peripheral edema (51% of patients), nausea (45%), and vomiting (28%). Twenty-three percent of patients experienced an AE that led to dose reduction, while AEs leading to treatment discontinuation were experienced by 15% of patients. The study also included exploratory patient-reported outcomes (PRO). The PRO analysis showed clinically relevant improvements in cough and preservation of patient quality of life (QoL) with capmatinib treatment [24].

As capmatinib has only recently been adopted in clinical practice and patients with advanced *MET*ex14 skipping NSCLC are few, real-world evidence on its use remains limited [25–27]. More robust data from various European countries could benefit practitioners. As such, this retrospective analysis aimed to evaluate data from patients with advanced *MET*ex14 skipping NSCLC who received compassionate use treatment with capmatinib in Italy before the AIFA approval.

## Methods

This was a retrospective analysis using real-world data from patients in Italy with advanced *MET*ex14 skipping NSCLC who received compassionate use treatment with capmatinib. The compassionate use provided access to capmatinib treatment for adult patients with metastatic *MET*ex14 skipping NSCLC, based on the US FDA’s accelerated approval in May 2020.

To receive compassionate use treatment with capmatinib, which was provided free of charge by the manufacturer, an unsolicited request from the treating physician for a patient was required. Stringent criteria were applied and patients were required to be assessed as having a serious or life-threatening disease or condition, with no comparable or satisfactory alternative therapies available. Furthermore, patients who received compassionate use treatment were not eligible for enrollment in a clinical trial.

This analysis is focused on Italian patients who received capmatinib as monotherapy in the second or subsequent treatment lines, after previously receiving immunotherapy and/or platinum-based chemotherapy for NSCLC.

The treating physicians obtained written informed consent, related to the treatment in compassionate use and the processing and use of data, from all participants or their representatives prior to the start of treatment, in accordance with the local laws and regulations and in line with the ethical principles outlined in the Declaration of Helsinki. As per the local laws and regulations (DECREE 17A07305 [GU Serie Generale n.256 del 02-11-2017]), ethics approval was not required for this retrospective analysis.

### Treatment

Capmatinib treatment was administered orally at a dose of 400 mg twice daily, with or without food. Compassionate use treatment continued until unacceptable toxicity, disease progression, treatment discontinuation (at the discretion of the treating physician), withdrawal of consent, or until treatment reimbursement approval by AIFA in May 2023.

### Data collection

Data were extracted from the manufacturer’s database and included data collected for each patient from the start of treatment with capmatinib as compassionate use up to April 30, 2023 (cut-off date). The collected data were kept confidential and were treated under the applicable Italian laws and regulations. The following data were extracted for this analysis: demographic characteristics, clinical characteristics (time since diagnosis, comorbidities, and sites of metastases), data confirming the presence of *MET*ex14 skipping mutations, previous NSCLC therapies received, dose of capmatinib and dose adjustments, treatment-related AEs, time to treatment discontinuation, and reasons for discontinuation.

### Statistical analysis

The analysis included all patients who received at least one dose of capmatinib and had received previous treatment with immunotherapy and/or platinum-based chemotherapy for NSCLC. Data from all centers were pooled and summarized. All statistical analyses were descriptive in nature. Continuous data were summarized using medians and ranges (minimum and maximum). Categorical data were summarized by absolute and relative frequencies (*n* and %) or contingency tables. The median time to treatment discontinuation and its associated 95% CI were evaluated using the Kaplan–Meier method. All statistical analyses were performed using the SAS software 9.4 (SAS Institute, Cary, NC, USA).

## Results

### Patient characteristics

A total of 112 requests for inclusion in the compassionate use program were submitted, and 84 patients received at least one dose of capmatinib. Among these, 28 were treatment-naïve (i.e., had not received prior therapy for advanced/metastatic disease), while 56 had undergone previous treatment. Of the pretreated patients, 53 had received capmatinib after prior treatment with immunotherapy and/or platinum-based chemotherapy; these individuals were the focus of the present analysis (Table 1). The observed population had a slight predominance of male patients (52.8%) and had a median age of 74 years at treatment initiation. Median age at NSCLC diagnosis was 72 years. The majority of patients (92.5%) had metastatic NSCLC, and most of the remaining patients (5.7%) had locally advanced disease. Only 22.4% of patients with metastatic disease had a single affected site, while 57.1% of patients had three or fewer metastatic sites (Table 1). The most common location for metastases was the lung (61.2%), followed by bone (40.8%) and the brain (22.4%; Table 1).

**Table 1:** Demographic and clinical characteristics of the study population

Characteristic	*n* = 53
*Sex, n (%)*	
Female	25 (47.2)
Male	28 (52.8)
Age at diagnosis, median (range), years	72 (51–88)
*Age at diagnosis according to age category, n (%)*	
< 75 years	33 (62.2)
≥ 75 years	18 (34.0)
Missing	2 (3.8)
Age at treatment start, median (range), years	74 (54–88)
*Age at treatment start according to age category, n (%)*	
< 75 years	27 (50.9)
≥ 75 years	26 (49.1)
*Disease stage at diagnosis, n (%)*	
Locally advanced	3 (5.7)
Metastatic	49 (92.5)
Unknown	1 (1.9)
Number of metastatic sites, *n* (%)	*n* = 49
< 3	28 (57.1)
≥ 3	20 (40.8)
Unknown	1 (2.0)
*Location of metastasis, n (%)*^*a*^	
Lung	30 (61.2)
Bone	20 (40.8)
Brain	11 (22.4)
Liver	8 (16.3)
Unknown	20 (40.8)
*PD-L1 TPS expression, n (%)*	
< 50%	15 (28.3)
≥ 50%	28 (52.8)
Unknown	10 (18.9)
Time from diagnosis to capmatinib start, median (range), months	14 (1–103)
*Number of prior treatment lines, n (%)*	
1	35 (66.0)
2	15 (28.3)
3	3 (5.7)
*Prior treatment*^*b*^	
Immune checkpoint inhibitors	19 (35.8)
Chemotherapy	17 (32.1)
Chemotherapy + immunotherapy	13 (24.5)

PD-L1, Programmed death-ligand 1; TPS, Tumor proportion score

^a^Patients could indicate more than one metastatic site

^b^Only the three most common prior treatments are listed. Prior treatment included monotherapy with immune checkpoint inhibitors and/or platinum-based chemotherapy

### Previous treatments

Patients started treatment with capmatinib after the failure of previous lines of treatment. Most patients (66.0%) had previously received one line of treatment, while 28.3% and 5.7% had already received two and three prior lines of treatment, respectively (Table 1). Previous treatments included immunotherapy and/or platinum-based chemotherapy.

### Treatment with capmatinib

Eligibility for capmatinib treatment was assessed by detecting *MET*ex14 skipping mutations, as per clinical practice guidelines.

Next generation sequencing (NGS) was the most frequently used technique for tumor molecular profiling (69.8% of patients). Other methods of detection included real-time polymerase chain reaction and immunohistochemistry.

The median time from diagnosis of locally advanced/metastatic NSCLC to initiating capmatinib was 14 months (Table 1). Most patients (98.1%) started treatment at the full recommended dose of capmatinib (400 mg twice daily), and more than 20% of patients required at least one dose reduction (Table 2). However, a single dose reduction was sufficient to optimize treatment in 91.7% of the patients requiring dose adjustments.

**Table 2:** Summary of exposure to capmatinib

Treatment summary	*n* = 53
*Capmatinib initial dose, n (%)*	
400 mg	1 (1.9)
800 mg	52 (98.1)
*Capmatinib dose adjustments, n (%)*	
No dose reduction	41 (77.4)
Dose reduction	12 (22.6)
1 dose reduction^a^	11 (91.7)
2 dose reductions^a^	1 (8.3)

^a^Percentage calculated considering patients who had ≥ 1 dose reduction (*n* = 12)

**Fig. 1:**
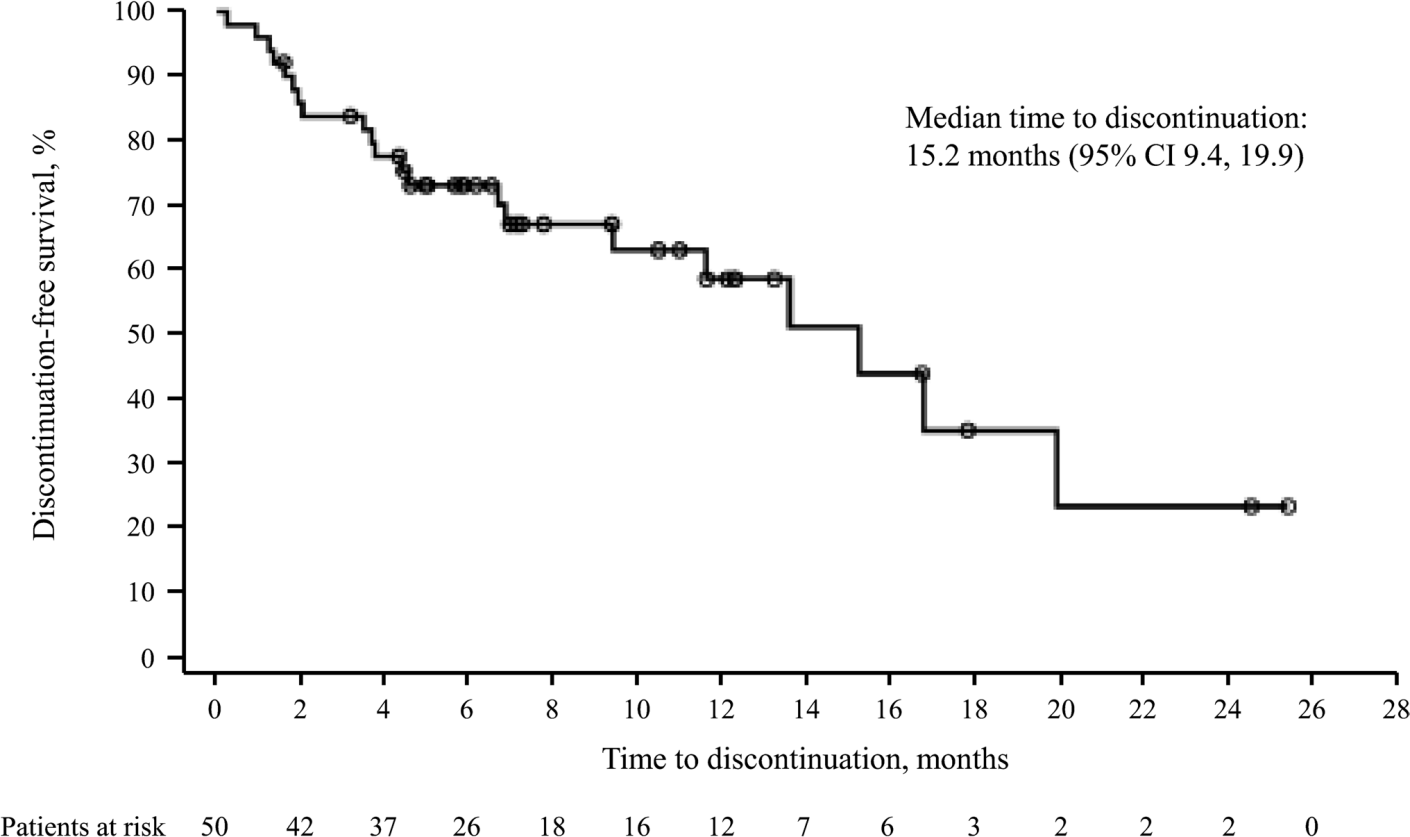
Kaplan–Meier estimate for the time to capmatinib discontinuation. Three patients were excluded from the analysis since time to event could not be computed due to unknown treatment end date. Dots represent censored patients. The time to event/censoring was calculated in months from treatment start date to date of event (i.e., treatment discontinuation) or date of censoring (i.e., cut-off date = April 30, 2023). CI, confidence interval

Median exposure to capmatinib was 6.36 months (range 0.3–25.4). Treatment with capmatinib was discontinued in 24 patients (45.3%). Capmatinib was discontinued due to disease progression (*n* = 11/24; 45.8%), AEs (*n* = 5/24; 20.8%), death (*n* = 4/24; 16.7%), or patient decision (*n* = 1/24; 4.2%). The reason for treatment discontinuation was unknown for 3 patients (12.5%). Kaplan–Meier analysis estimated a median time to treatment discontinuation of 15.2 months (95% CI 9.4, 19.9) (Fig. 1).

## Discussion

Owing to the discovery of several molecular-defined subgroups of NSCLC and the availability of several treatment options for these subgroups, which are associated with improved outcomes and patient QoL, oncologists face new challenges when selecting the most appropriate therapy. Molecular characterization for each patient should be available while proper treatment selection and treatment sequencing customization are still a matter of debate.

Due to the rarity of *MET*ex14 skipping mutations in NSCLC and the typically older age of the population carrying this mutation, the enrollment of large numbers of patients in clinical trials evaluating targeted therapies is challenging. In addition, studies in the *MET*-mutated NSCLC population are lacking because the *MET*ex14 skipping mutation has only recently been identified as a predictive biomarker [28–30].

Tumor molecular profiling is a mandatory component of treatment decisions for advanced NSCLC. Owing to the complexity and variety of actionable genomic alterations in NSCLC, the use of NGS is now widely recommended by international guidelines [1, 5]. Our report shows that NGS was used in most cases (69.8% of patients); this could be improved, for example by increasing access to NGS and knowledge of the latest molecular testing techniques. As *MET*ex14 skipping mutations have been only recently recognized as biomarkers and can be difficult to detect due to the type of alteration, assays for their detection by NGS and other techniques still need to be optimized [21]. Ideally, an RNA-based panel should be used for detection of *MET*ex14 skipping mutations by NGS, but this is associated with some technical difficulties [31].

In this retrospective analysis, we described the experience of 53 patients with advanced/metastatic *MET*ex14 skipping NSCLC who were treated with capmatinib, within a compassionate use program after the failure of one or more lines of standard therapy (platinum-based chemotherapy, immune checkpoint inhibitors, or the combination of both). The treated population was characterized by advanced age and the presence of several metastatic sites, including brain metastases in > 20% of patients. Despite the expected frailty of this population, treatment with capmatinib was associated with a prolonged retention and an estimated median time to discontinuation of more than 15 months. Treatment was discontinued mostly due to disease progression, while AEs led to treatment discontinuation in only 5 patients (9.4% of the total population). In contrast, in the capmatinib registration trial GEOMETRY mono-1, which enrolled 160 patients (mean age of 71.3 years; 61% female; 85% aged ≥ 65 years) with *MET*ex14 NSCLC (100 of whom were receiving capmatinib as second- or later-line treatment), 20% of patients discontinued treatment due to AEs [23]. In this study, the median duration of exposure to capmatinib was 34.9 weeks (interquartile range 13.0–80.6; 8.03 months; 2.99–18.55).

Attempts to characterize NSCLC patients carrying *MET*ex14 skipping mutations have been reported in the literature [32, 33]. A systematic review of the literature about frequency, characteristics, and outcomes of patients with these mutations identified only advanced age, adenocarcinoma histology, and a poor prognosis to be distinctive characteristics of *MET*ex14 skipping-mutated NSCLC patients [33]. Objective response rates (ORR) to treatments in these patients range from 50 to 70% with targeted therapies, 33% with immunotherapy, and 23–27% with chemotherapy.

A few reports specifically describing real-world experiences with capmatinib in NSCLC have been published [25, 26, 34]. The international RECAP retrospective study included 81 patients with NSCLC and *MET*ex14 skipping mutations who received capmatinib as first- and later-lines [25]. In this study, molecular profiling was performed mainly by NGS. Consistent with our findings, the median age of patients was advanced (77 years), most patients (86%) had metastatic disease, and 27% had brain metastases. The median follow-up was 10.7 months. As in our observations, treatment was discontinued mostly due to disease progression (61%), while AEs and death accounted for 24% and 10% of discontinuations, respectively. Another real-world study analyzed 68 patients (median age 64.6 years) with *MET*ex14 skipping NSCLC and brain metastases who were treated with capmatinib in US routine clinical practice [26]. This study was prompted by the encouraging findings from the GEOMETRY mono-1 study, which suggested that capmatinib was associated with intracranial lesion shrinkage in patients with brain metastases [23]. In this study, after a median duration of follow-up of 8.2 months from the start of capmatinib therapy, treatment discontinuation rates were 18.2% and 23.1% for first- and second-line capmatinib, respectively [26]. It is important to note that these real-world studies, which included first-line treatment cohorts, are in contrast with the current EMA and AIFA indications for capmatinib that require patients to have previously received treatment with immunotherapy and/or platinum-based chemotherapy [17, 22].

Kron and colleagues conducted a retrospective analysis of the selective use of capmatinib versus standard of care (SOC) for NSCLC patients with *MET*ex14 mutations in German routine care, including data from patients enrolled in the GOEMETRY mono-1 study and patients analyzed in the RECAP study [35]. The results of this analysis further emphasized the robust clinical benefit of capmatinib for patients with NSCLC harboring *MET*ex14 mutations compared with SOC, particularly in preventing brain metastases. However, the authors highlighted the need for real-world data to confirm the implications of the availability of capmatinib in clinical practice, a finding that was further supported by Sini and colleagues in a letter in response to this publication who emphasized some limitations of this analysis [36].

The safety and tolerability of treatments are particularly important in the management of patients with *MET*ex14 skipping NSCLC due to the advanced age of the patients harboring this alteration. Evidence from the GEOMETRY mono-1 study has shown a relatively manageable profile of AEs, with peripheral edema being the most frequently reported event [26]. For patients experiencing ≥ Grade 3 edema with capmatinib treatment, temporary dose interruption is recommended [37]. As previously pointed out, the safety profile of capmatinib appears to be confirmed also in the real-world setting [25, 26]. Extensive post-marketing safety monitoring of capmatinib is ongoing and will likely help define the position of this new treatment option for NSCLC [38, 39].

Our analysis has several limitations including the small sample size and the retrospective nature of the analysis. Furthermore, as capmatinib was provided as part of a compassionate use, safety, and efficacy data were not collected due to the inability to follow patients beyond the treatment period. However, we believe that, despite these limitations, our report provides a glimpse into real-world management of NSCLC treatment with capmatinib in Italy. In particular, time to treatment discontinuation is a useful surrogate to determine the clinical significance of a treatment in real-world practice, as it highlights actual clinical use of the drug, including beyond progression.

## Conclusion

This real-world retrospective analysis confirms the clinical characteristics of patients with *MET*ex14 skipping NSCLC and highlights that capmatinib is a tolerated and valuable treatment option in this difficult-to-treat population. Despite having poor prognostic factors, such as brain metastases and advanced age, our patients tolerated capmatinib effectively and continued treatment over a prolonged duration, largely at the original full dosage. Evidence generated from real-world data makes an important contribution to the evidence obtained from clinical trials in thoroughly selected patients, and may be transferred to a broader patient population.

## Data Availability

The datasets generated during and/or analyzed during the current analysis are available on reasonable request. For original data, please contact Ilaria_gioia.marcon@novartis.com and diletta.valsecchi@novartis.com.
