# Correction: Capmatinib for the treatment of *METex*14 skipping non-small cell lung cancer: retrospective analysis of real-world data from patients receiving compassionate use treatment in Italy

**DOI:** 10.1007/s10238-025-01988-2

**Published:** 2025-12-18

**Authors:** Fabiana Letizia Cecere, Ettore D’Argento, Francesco Gelsomino, Francesco Pesola, Paolo Bironzo, Salvatore Grisanti, Laura Bonanno, Gianluca Spitaleri, Diletta Valsecchi, Ilaria Marcon, Diego Cortinovis

**Affiliations:** 1 Medical Oncology 2, IRCCS Regina Elena National Cancer Institute Rome, Rome, Italy; 2 Comprehensive Cancer Center, Medical Oncology Department, Fondazione Policlinico, Universitario Agostino Gemelli IRCCS, Rome, Italy; 3 Medical Oncology, IRCCS Azienda Ospedaliero-Universitaria di Bologna, Bologna, Italy; 4 Thoracic Oncology Unit, IRCCS Istituto Tumori “Giovanni Paolo II”, Bari, Italy; 5 Department of Oncology, University of Torino, AOU S. Luigi Gonzaga, Orbassano, Italy; 6 Medical Oncology, ASST Spedali Civili di Brescia, University of Brescia, Brescia, Italy; 7 Department of Surgery, Oncology and Gastroenterology, University of Padova, Padua, Italy; 8 Medical Oncology 2, Istituto Oncologico Veneto IOV I.R.C.C.S, Padua, Italy; 9 Thoracic Oncology Division, European Institute of Oncology IRCCS, Via Ripamonti, 435, 20141 Milan, Italy; 10 Novartis Farma SpA, Milan, Italy; 11 Medical Oncology Fondazione, IRCCS San Gerardo dei Tintori Monza, Via Pergolesi 33, 20900 Monza, Italy; 12 Medicine Department University Milano-Bicocca, Milan, Italy

**Correction to: Clinical and Experimental Medicine (2025) 26:18** 10.1007/s10238-025-01934-2

In the original version of the article the affiliation details for Author Gianluca Spitaleri were incorrectly given as

Thoracic Oncology Division, European Institute of Oncology, Via Ripamonti, 435, 20141 Milan, Italy

But should have been

Thoracic Oncology Division, European Institute of Oncology IRCCS, Via Ripamonti, 435, 20141 Milan, Italy

